# Isolation and characterization of highly pathogenic avian influenza virus subtype H5N1 from donkeys

**DOI:** 10.1186/1423-0127-17-25

**Published:** 2010-04-14

**Authors:** Ahmed S Abdel-Moneim, Ahmad E Abdel-Ghany, Salama AS Shany

**Affiliations:** 1 Department of Virology, Faculty of Veterinary Medicine, Beni-Suef University, Beni-Suef 62511, Egypt; 2 Division of Virology, Department of Microbiology, College of Medicine and Medical Sciences, Taif University, Al-Taif, Saudi Arabia; 3 Department of Hygiene, Management & Zoonoses, Faculty of Veterinary Medicine, Beni-Suef University, Beni-Suef 62511, Egypt; 4 Department of Poultry Diseases, Faculty of Veterinary Medicine, Beni-Suef University, Beni-Suef 62511, Egypt

## Abstract

**Background:**

The highly pathogenic H5N1 is a major avian pathogen that crosses species barriers and seriously affects humans as well as some mammals. It mutates in an intensified manner and is considered a potential candidate for the possible next pandemic with all the catastrophic consequences.

**Methods:**

Nasal swabs were collected from donkeys suffered from respiratory distress. The virus was isolated from the pooled nasal swabs in specific pathogen free embryonated chicken eggs (SPF-ECE). Reverse transcriptase polymerase chain reaction (RT-PCR) and sequencing of both haemagglutingin and neuraminidase were performed. H5 seroconversion was screened using haemagglutination inhibition (HI) assay on 105 donkey serum samples.

**Results:**

We demonstrated that H5N1 jumped from poultry to another mammalian host; donkeys. Phylogenetic analysis showed that the virus clustered within the lineage of H5N1 from Egypt, closely related to 2009 isolates. It harboured few genetic changes compared to the closely related viruses from avian and humans. The neuraminidase lacks oseltamivir resistant mutations. Interestingly, HI screening for antibodies to H5 haemagglutinins in donkeys revealed high exposure rate.

**Conclusions:**

These findings extend the host range of the H5N1 influenza virus, possess implications for influenza virus epidemiology and highlight the need for the systematic surveillance of H5N1 in animals in the vicinity of backyard poultry units especially in endemic areas.

## Background

Influenza A viruses belong to the family *Orthomyxoviridae *and have been isolated from a variety of different species. Further subtyping of influenza A viruses is based on antigenic differences between the two surface glycoproteins haemagglutinin (H1-H16) and neuraminidase (N1-N9) of the influenza A viruses [1,2]. The HA mediates the attachment of the virus to sialic-acid-containing receptors on the host cell surface, as well as the fusion of the virus envelope with the cellular membrane [3,4]. The specificity of the HA towards these molecules differs. Avian and equine influenza viruses preferentially bind the sialic acid α-2,3-galactose (SAα2,3Gal) linkage, while human influenza viruses preferentially bind the SAα2,6Gal linkage [5-7]. The highly pathogenic avian influenza virus H5N1 (HPAIV- H5N1) represents an important poultry pathogen and a major havoc to the poultry industry. Furthermore, HPAIV H5N1 infections in poultry constitute a threat to mammals including humans. Apart from humans, the natural infections of H5N1 influenza A have been reported in several mammalian species including domestic cats [8], tigers and leopards [9], dogs [10], pigs [11] and stone marten [12]. Experimentally, H5N1 has also been able to infect mice [13], ferrets [14], monkeys [15] and cattle [16]. Infections in either experimental or naturally infected hosts have been fatal except for pigs and cattle where mild to subclinical infections have been detected [11,16]. No naturally occurring cases of H5N1 HPAI have been reported in horses or other members of the *Perissodactyla *order nor have any experimental studies been published [17]. An equine influenza virus;H3N8 with avian gene pool has been isolated emphasizing that equines may be susceptible to avian influenza viruses of the H3N8 subtype[18] and possibly others. The family *Equidae *and in particular donkeys may be of great importance in certain endemic countries like Egypt where they are commonly housed together with poultry. Long term endemic influenza virus infections in poultry increase exposure risks to surrounding humans and other mammals and in turn, create opportunities for the emergence of human-adapted strains with pandemic potential [19,20]. Since 2006, H5N1 influenza A virus has been endemic in Egypt producing great economic losses and most importantly hitting humans hard with high case fatality rate; 34/109 (WHO; http://www.who.int/csr/disease/avian_influenza/country/cases_table_2010_04_09/en/index.html).

Here we report the isolation of HPAI H5N1 from donkeys living in contact with diseased birds and demonstrate the presence of H5 seropositive ones in the neighbouring areas.

## Methods

### Virus isolation

Nasal swabs were collected from three infected animals from Aborady village, El-Wasta locality, Beni-Suef Governorate. Each swab was placed in a tube containing 0.5 ml sterile normal saline containing gentamicin sulfate solution (50 mg/ml). The swab tip was cut off in the saline and the tubes were immediately transported to the lab for testing in an ice box to be processed using a routine method. Infected materials were pooled, centrifuged at 500 x g for 10 min. and then inoculated into the allantoic cavity of five, 10-day-SPF-ECE (100 μl/egg). Inoculated embryos were incubated at 37°C for 24-48 h.

### Haemagglutination inhibition

One hundred and five serum samples were collected from apparently healthy donkeys from different localities in the Beni-Suef Governorate, 4-6 months after the procedure of virus isolation. Sera were heat inactivated for 30 min at 56°C and 2-fold serial dilutions were performed in 25-μL volume in 96-well HI plates. Equal volumes of 4HA of H5 influenza virus antigen (A/chicken/Egypt/F6/2007(H5N1)) were added to diluted serum samples then 1% suspension of human erythrocytes were dispensed to each well [21]. HI titers ≥ 3 log_2 _were considered positive. Samples assayed in duplicates and each assay was validated by comparison with positive and negative chicken and equine control sera as well as back titration of the used virus dilutions.

### Viral RNA extraction and RT PCR

Viral RNA was extracted from virus containing chorioallantoic membranes (CAM) homogenate by using a SV Total RNA Isolation System (Promega Corporation, Madison, Wis.). The sample was processed alone in a sterile clean room to avoid the possibility of any cross contamination. One-step RT-PCR amplification for full length of both NA and HA genes were performed using Verso™ 1 step RT PCR (Thermo Fisher Scientific Inc.). A single set of primers was used for NA (For:ATGAATCCAAATCAGAAG, Rev:TGTCAATGGTGAATGGCAAC) but for HA, four sets of primers flanking overlapping regions of the full length gene were used (Primer sequences for HA were ordered according to that provided by; Laboratory for Molecular and Biological Characterization of AIV, FLI, Germany).

### Sequence analysis

Amplicons were first subjected to 1% gel electrophoresis and specific bands were excised and purified using EzWayTM gel extraction kit (Komabiotech, Korea). Each purified amplicon was sequenced in both forward and reverse directions (Macrogen Inc., Korea).

### Phylogenetic Analysis

BLAST analyses were initially performed to establish HA and NA sequence identities to GenBank accessions [22]. Comparative analyses were performed using the CLUSTAL W Multiple Sequence Alignment Program, Mega 3.1. AIV representative sequences used for the alignments were obtained from the GenBank and EMBL database. The phylogenetic trees were constructed by using the neighbour-joining method with Kimura two-parameter distances by using MEGA version 3.1 [23]. The reliability of internal branches was assessed by 1000 bootstrap replications and p-distance substitution model.

## Results and discussion

**Figure 1:**
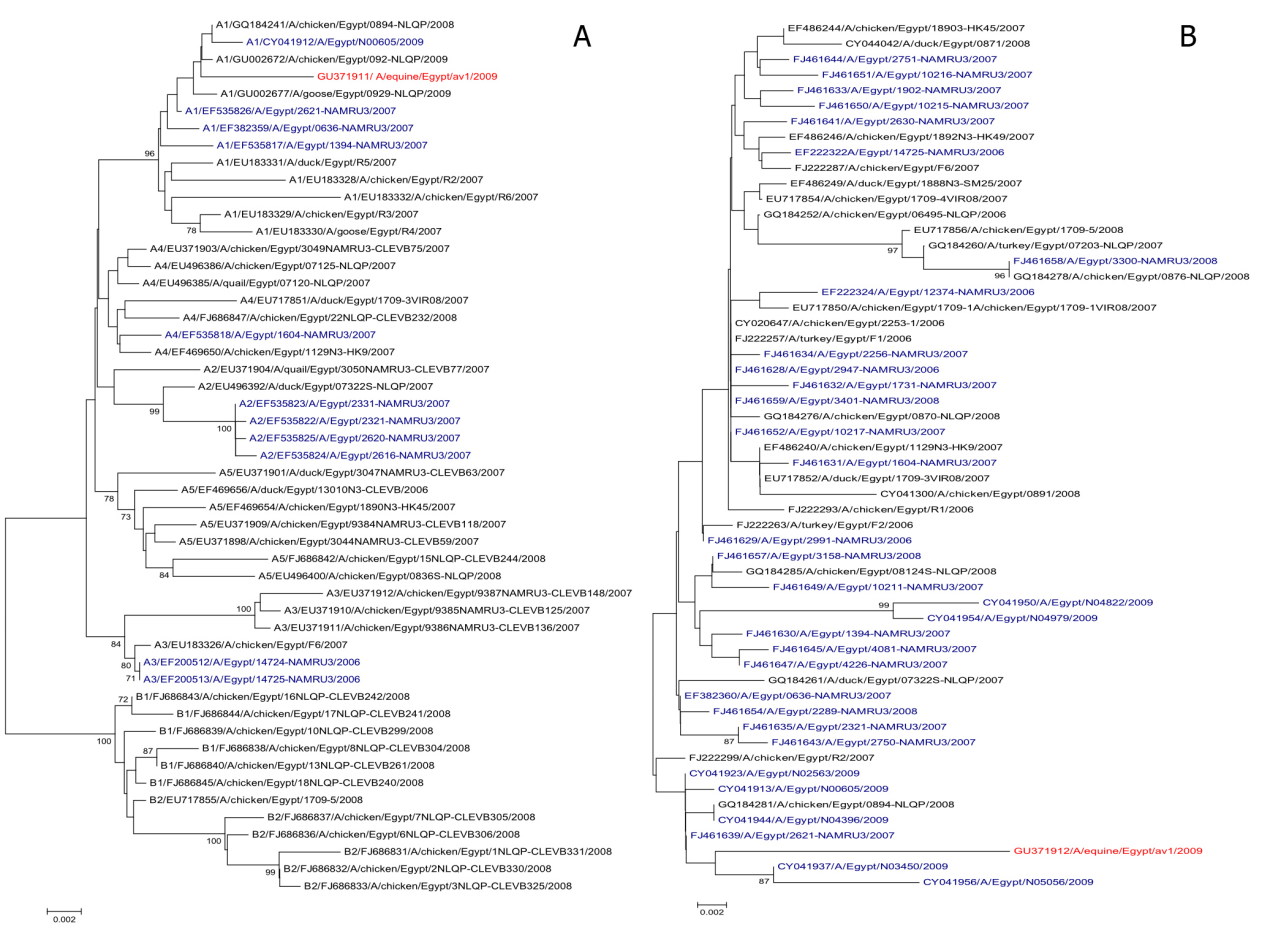
**Phylogenetic analyses of HA and NA of an equine H5N1 isolate sequence in comparison to Egyptian human and avian isolates**. a, HA gene b, NA gene. Human isolates are shown in blue while avian ones are shown in black whereas equine isolate is shown in red. All sequences were obtained from GenBank. Trees were generated using Neighbour-Joining method. The robustness of individual nodes of the tree was assessed using a bootstrap of 1000 resamplings in per cent (70% and higher) are indicated at key nodes.

**Figure 2:**
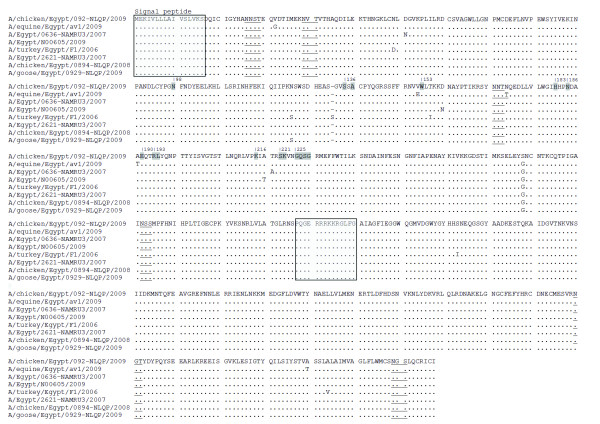
**Deduced amino acid sequences of the HA protein of equine H5N1 isolate in comparison to closely related Egyptian HPAIV H5N1 isolates**. Dots denote identical amino acids, which are given in one-letter code. Consensus sequences for N-glycosylation (NXS or NXT, except where X = P) are underlined. Boxed segments indicate the signal peptide and the polybasic proteolytic cleavage motif, respectively. Shaded letters denote potential sites responsible for receptor binding sites (H3 influenza numbering).

In this study, we isolated H5N1 form donkeys clinically affected with moderate respiratory distress including cough, fever and serous nasal discharge. The course of the disease was short (72 h) and responsive well after two shots of streptomycin/penicillin antibiotic therapy and one shot of antipyretic (Diclofenac sodium) with no recorded mortalities. The inhibition induced by antibiotic to the possible secondary bacterial invasion, besides the moderate severity of the H5N1 in donkeys may be responsible for the recovery of the infected animals without further complications. The disease recoded on 24^th ^March 2009, 1 wk after an outbreak of H5N1 infection in poultry in the village, where many donkeys suffered from the same clinical manifestations in an epidemic manner. The virus was isolated from a pool of nasal discharge from three affected animals. It produced haemagglutination only after the 3^rd ^egg passage. RT-PCR was performed to the full length of both NA and HA genes, where they were sequenced directly after gel purification. Sequences were deposited in GenBank under accession numbers; GU371911 and GU371912 for HA and NA respectively. The HA and NA genes of the investigated equine isolates revealed that they belonged to (5J), (1J) lineages respectively (According to the Influenza A Virus Genotype Tool) [24]. Phylogenetic analyses revealed that the HA of the equine isolate related to sublineages, A (A1) (Fig. 1). The equine isolate showed a typical polybasic cleavage motif with the GERRRKKR*GLF consensus sequence found in clade 2.2 viruses. It also contains amino acid D403 characteristic to sub-clade 2.2.1 (Fig. 2). The haemagglutinin gene was found to be closely related to A/chicken/Egypt/0894-NLQP/2008, A/Egypt/N00605/2009 and A/chicken/Egypt/092-NLQP/2009 while the neuraminidase gene of the current strain is closely related to A/Egypt/N03450/2009 and A/Egypt/N05056/2009. However, none of these strains were isolated from localities near to Beni-Suef.

The equine isolate has amino acids Q226 and G228 (H3 influenza numbering) denoting the preferential binding of α-2,3 linkage, typical for the avian and equine viruses but not human ones [5,25]. This finding demonstrated that the isolates with avian specific receptor binding properties can replicate and cause infection in equines. Different amino-acids that are implicated in receptor specificity Y98, S136, W153, H183, E190, K193 L194 E216 P221 K222 G225, Q226, S227, G228 (H3 influenza numbering) were tested [26]. Interestingly 98 (Y to N), 193 (K to R), 216 (E to K) and 221(P to S) which were found in the examined equine isolate have also been in many other isolates from the middle east in the flu database which raises a question as to what the impact of such substitution on HA binding to human receptors is. Recently, A138V, N186K and S227N mutations were reported to confer α-2,6-linked sialic acid binding to H5N1 virus [27-30]. None of such substitutions were found in the equine isolate. The HA of the human or avian Egyptian isolates, contains a total of seven potential N-glycosylation signals whereas the equine isolate contains an additional N-glycosylation (Fig. 2).

In addition, the equine isolate lacks aa S145. This deletion is also present in all other viruses grouped into 2.2 sublineage A1, which also includes sequences from human H5N1 isolates (Fig. 2). The significance of this deletion is unknown, but it should be noted that this position is close to a domain modulating receptor interaction. Interestingly, strains with this deletion appear to evolve towards a receptor usage that is similar to that of the seasonal human H1N1[31].

**Figure 3:**
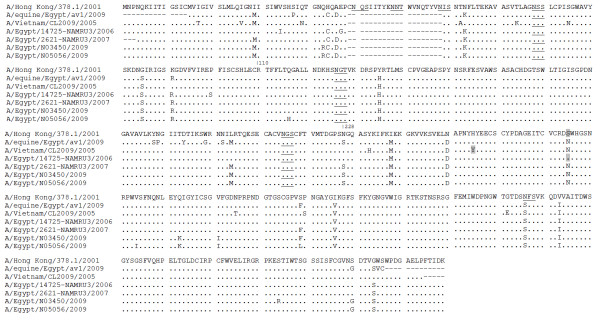
**Deduced amino acid sequences of NA protein of equine H5N1 isolate in comparison to two recent closely related and other distant isolates that showed resistant to oseltamivir**. Dots indicate residues identical amino acids. Underlined letters are N-glycosylation sites, shaded letters showed site of H274Y and N294S substitutions (H275Y and N295S in N1 influenza numbering).

Analysis of the NA gene revealed the presence of the 20-amino acid deletion (Fig. 3) as well as the presence of amino-acid R at position 110 which is characteristic of clade 2.2 viruses [32]. Three potential N-glycosylation sites were predicted (Fig. 3). The 228 (N to S) substitution is also present and indicative to 2.2.1 virus (2009). Four NA mutations; E119G, H274Y, R292K, and N294S have been reported to confer resistance to NA inhibitors [33] but none were detected in the equine isolate.

**Figure 4:**
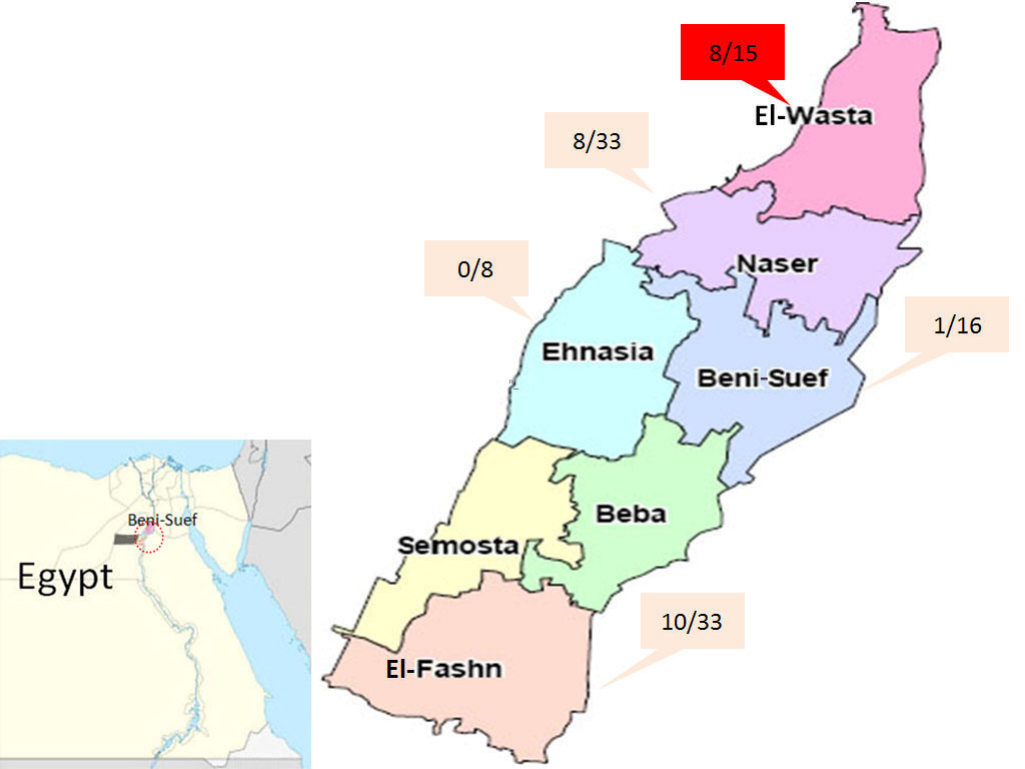
**Beni-Suef map, showed the distribution of H5 seropositive equine samples (donkeys)**. HI test was performed using local Egyptian antigen (A/chicken/Egypt/F6/2007), red box denotes the locality where the A/Egypt/equine/av1/2009 was isolated.

Speculating that infected animals may mount an antibody response depending on the interval post infection, we screened H5-specific antibodies 4-6 months after the initial virus isolation. The H5 specific antibodies were detected in naturally affected animals. 27 out of 105 (25.71%) of the examined animals were H5 positive with the highest percentage found in the area where the virus was isolated (Table 1, Fig. 4).

**Table 1:** Serological screening of H5N1 exposure in donkeys from different localities of Beni-Suef Governorate using haemagglutination inhibition assay

Locality	Samples				HI range (Log_2_)
		
	Number	Positive	Negative	Positive %	
Beni-Suef	16	1	15	6.25	7
El-Fashn	33	10	23	30.30	8-10
Ehnasia	8	0	8	00.00	0-2
El-Wasta	15	8	7	53.33	6-9
Naser	33	8	25	24.24	7-8
Total	105	27	78	25.71	

## Conclusion

We did note the incidence of clinical infections of donkeys with HPAIV (H5N1) in disease endemic regions where the probability of intimate contact between poultry and donkeys is high. Furthermore, H5 seroconversion by naturally exposed donkeys was evidenced. Although the disease did not constitute a real threat to donkeys, it raises the concern of different issues including the route of transmission to donkeys, whether being from aerosol exposure of pulverized infected birds droppings or contaminated feeds and water or because of contact with infected birds. Second, the role of donkeys in spreading H5N1 virus to birds, humans and other mammals including equines needs to be assessed.

## Competing interests

The authors declare that they have no competing interests.

## Authors' contributions

ASA and AEA designed, performed experiments, and analyzed data. ASA generated genetic constructs and drafts the manuscript. AEA and SASS performed the HI analyses and helped in RT-PCR. AEA reviewed the manuscript.
